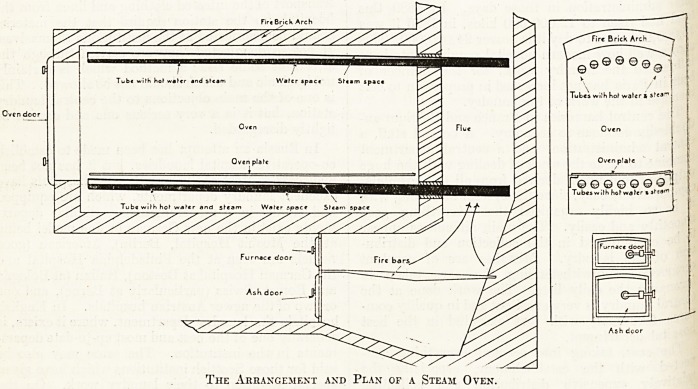# Baking Bread in a Steam Oven

**Published:** 1913-02-01

**Authors:** 


### PRACTICAL POINTS.

(Criticism and Suggestions Invited.)

### Baking Bread in a Steam Oven (*Illustrated*).

The steam oven is by no means new. It is claimed by Mr. Jacob Perkins that he invented the steam pipe, upon which the working of the steam oven depends, some forty years ago. The arrangement of a steam oven itself is an adaptation of the principle of the delivery of heat by steam, a very convenient vehicle for this purpose. With the steam-heated baker's oven there is really no steam boiler, as it ordinarily exists in appliances for distributing heat by means of steam. Instead, there are a number of steam pipes, each of which forms a little boiler in itself. The oven is constructed very much in the usual way, lined with brick.

The object of this is to absorb as much heat as possible from the steam pipes and to keep the temperature throughout the oven as uniform as possible. There is the usual hot plate, upon which the loaves to be baked are placed. Under the hot plate is a row of steam pipes closed at each end. One end of each of the steam pipes projects for a short distance through the end wall of the oven into the furnace. There is another row of steam pipes fixed under the brick arch which forms the top of the oven. The ends of these pipes are also closed, and one end of each projects through the end wall into the flue above the furnace.

Each pipe contains a small quantity of water; when the oven is cold the water rests in the ends of the pipes which are within the furnace chamber and the flue above. The pipes are at a slight inclination, so that any water which is present will run down to the furnace chamber. When the oven is cold there is only water in the pipes, and a certain quantity of the vapour of water. When the fire is lighted on the grate in the furnace chamber the water in the pipes is first heated, expands, and is gradually formed into steam. The steam has its temperature gradually raised to somewhere in the neighbourhood of 550° to 600° F. Heat passes from the steam pipes through the hot plate directly to the loaves, and a smaller quantity of heat also passes from the steam pipes above to the upper parts of the loaves. The brickwork absorbs heat from the upper steam pipes, and the whole oven assumes the uniform temperature that is so advantageous for good baking. The arrangement is shown in the illustration. There is a thermometer attached to the oven, the scale of which is fixed in front, graduated up to 600° F., and usually marked with a red line at 500° F. From 450° F. to 500° F. has been found by long experience to be the best temperature for producing a good quality of bread.

The bulb of the thermometer is inside the chamber, and is connected with the scale by a tube of metal. There is also a damper fixed in the flue, by means of which the baker in charge of each oven can regulate the draught passing through the furnace, and by so doing he can regulate the quantity of heat delivered to the steam pipes and the temperature within the oven. By opening or closing the damper and by carefully feeding the fire upon the grate a perfectly uniform temperature is easily maintained. Further, when other comestibles are to be baked, such as buns or pastry, requiring not such a high temperature as bread, it is quite easy to arrange that only the requisite temperature rules within the oven. The furnace of the oven is fired with coke or with anthracite coal, and it is claimed that the steam oven, worked upon the above principle, only consumes half the quantity of coke that the ordinary fire-heated oven would consume in coal. The steam oven, with its uniform arrangement of pipes, has the great advantage that the heat is far better distributed than is possible with the ordinary arrangement of fire-heating and brick flues. Coke or anthracite coal is employed because the ordinary bituminous coal gives rise to smoke, which deposits soot upon the ends of the steam pipes within the furnace chamber. Soot offers a resistance to the passage of heat from the hot gases of the furnace to the water or the steam inside the pipes. With coke there is no trouble of that kind. There is a certain deposit of soot upon the ends of the upper row of pipes, and soot doors are provided for cleaning them and the flue as a whole.

The Arrangement and Plan of a Steam Oven.

A complete outfit for baking a batch of loaves, such as would be required to supply a hospital, includes a sifter, through which the flour is passed to remove any foreign matter, such as fluff, small pieces of stick, or stone that have got in amongst the flour. It is found that foreign flours in particular are very liable to contain small quantities of "matter in the wrong place." From the sifter the flour passes into a kneading machine, which consists of a trough, in which two arms or blades are revolved by power; these perform in a few minutes the work that would take the housewife or the hospital baker at least half an hour. Water of the proper temperature, 90° F., and of the proper quantity is run into the trough from a tank, to which is attached a thermometer and a scale showing the quantity delivered. After kneading, the dough is turned out on to a trough, and, where large quantities are dealt with, passes successively through machines which cut it up into measured quantities and mould the tops and bottoms of the loaves ready to go into the oven.

## Figures and Tables

**Figure f1:**